# The Correlation of Temperature-Mineral Phase Transformation as a Controlling Factor of Thermal and Mechanical Performance of Fly Ash-Based Alkali-Activated Binders

**DOI:** 10.3390/ma13225181

**Published:** 2020-11-17

**Authors:** Natalia Kozhukhova, Marina Kozhukhova, Irina Zhernovskaya, Vladimir Promakhov

**Affiliations:** 1 Department of Material Science and Material Technology, Belgorod State Technological University named after V.G. Shukhov, Kostukov St., 308012 Belgorod, Russia; ziv_1111@mail.ru; 2 Department of Civil & Environmental Engineering, University of Wisconsin Milwaukee, 3200 N Cramer Str., Milwaukee, WI 53201, USA; kozhuhovamarina@yandex.ru; 3 Center of Additive Technologies, National Research Tomsk State University, 36, Lenin Ave., 634050 Tomsk, Russia; vvpromakhov@mail.ru

**Keywords:** alkali-activated binder systems, high-temperature alumosilicates, Class F fly ash, nanosized nepheline, thermal-resistant properties

## Abstract

This research focuses on an evaluation of mineral phase and structure transformations in Class F fly ash-based geopolymer systems. The research also studies the strength response of geopolymers when exposed to temperatures between 25 and 800 °C. The purpose of this research is to understand the processes that occur in alkali-activated systems within a wide range of high-working temperatures. The XRD, SEM, and DTA/TG analyses performed for the alkali-activated compositions after exposure to different temperatures confirmed a direct correlation of structural transformations with strength performance. The detrimental effect of sodium hydrocarbonate Na_3_(HCO_3_)(CO_3_) 2H_2_O or trona contained in one of the fly ash products was observed for the corresponding alkali-activated composite under high-temperature exposure between 600 and 800 °C. It was also detected that a high-temperature interval of 400–800 °C created favorable conditions that helped to form nanosized nepheline crystals and an additional vitreous substance that also contributed to a denser alkali-activated matrix.

## 1. Introduction

A constant increase in material volume production results in depletion of nonrenewable natural resources. This drives all sectors of the construction industry to look for alternatives such as industrial byproducts [1,2]. At the same time, there are enormous volumes of unutilized industrial byproducts such as coal ash which are disposed of in landfills and impoundments which have a negative impact on the environment of the adjacent territories. This environmental challenge, in combination with the constant development of infrastructure and growing demand for materials, makes the design of novel, efficient, and multifunctional construction materials using green and zero-waste technologies a hot topic in the construction industry worldwide.

The Convention on Climate Change founded under the Kyoto Protocol and responsible for the implementation of the programs in the waste management sector, came up with strict industrial emission limits for industrialized developed countries. In this regard, the utilization of industrial byproducts in construction materials is a primary goal of the construction industry in the XXI century [3].

Among all industrial byproducts that are currently being utilized, coal fly ash produced by power plants is in high demand in concrete manufacturing as a partial replacement for Portland cement in the form of supplementary cementitious material as well as pozzolanic admixture [4]. This is due to the positive effect that fly ash has on the cement systems such as a reduction in hydration heat, and therefore reduction in plastic deformation and premature formation of cracks in concrete. At the same time, pozzolanic properties of fly ash allow the formation of additional C-S-H gel at a later age of curing, which contributes to the densification of cement matrix, and therefore improves the long-term performance of concrete [4,5].

For the last few decades, there have been a number of studies which have focused on the development of blended cement systems [6,7] including alkali-activated systems based on soil cements [8], slags [9,10], and alumosilicates in the form of metakaolin [11].

For several decades, fly ash products have been demonstrated to be an excellent resource for zero-Portland cement binders [12,13,14]. Geopolymers or fly ash-based alkali-activated binding systems proposed by Davidovits provided a bright example of zero-Portland cement binder systems [15]. The high practical potential of geopolymers and their green production technology have been reported by multiple research studies [16,17].

In terms of chemical composition, geopolymers, like many other construction materials and binders, are mostly comprised of alumosilicates. However, regardless of the presence of the oxides SiO_2_ and Al_2_O_3_ in the composition, thermal resistant properties of materials and binders are essentially governed by both oxide and mineral composition and SiO_2_/Al_2_O_3_ (Si/Al) ratio.

Davidovits, in his work [18], reported that microstructure properties, as well as areas of application of low-Ca alumosilicate binders such as geopolymers, were tightly hinged upon the ratio of SiO_2_ and Al_2_O_3_, the main oxides in the system. For example, such construction materials as bricks, ceramics, and other fire protection materials have Si/Al ratios within the range of 1–2; and sealants for industry application, exposed to high temperatures from 200 °C to 600 °C, represent a group of materials with Si/Al > 3.

K. Hemra and P. Aungkavattana [19] reported that thermal resistance of metakaolin-based alkali-activated alumosilicate binder with Si/Al = 3.5 at a temperature as high as 800 °C could reach up to 15 cycles, in accordance with the standard ASTM C1171-16.

Additionally, the study by Bajare D. et al. [20,21] proved that, with Si/Al < 2 and an increase in the Al_2_O_3_ portion in the composition of alkali-activated binders, it resulted in a better response of thermal resistance in the temperature range of 800–1000 °C due to formation of high-temperature resistant mineral phases such as carnegeite and nepheline.

Other research studies [22,23] have reported that alkali-activated binders-based concrete and mortars exhibited superior refractory performance up to 1000 °C as compared with traditional Portland cement composites, which makes geopolymers a promising alternative for refractory materials production.

As noted above, alkali-activated binders attain thermal resistant properties due to specific alumosilicate oxides composition which is very similar to that in unburned ceramic materials such as highly concentrated binding systems (HCBS) invented by Yu. A. Pivinsky [24], and nanostructured binders developed by A.V. Cherevatova’s research group [25,26,27]. These materials reveal enhanced resistance to high temperature (refractory ceramics).

A number of studies related to phase formation in alkali-activated binders exposed to high temperatures [28,29] have concluded that the binder matrix produced by alkali activation of Class F fly ash predominantly composed of sodium alumosilicate hydrates (i.e., sodalite Na_8_(Al_6_Si_6_O_24_)·2H_2_O, anorthite Ca(Al_2_Si_2_O_8_), albite NaAlSi_3_O_8_), formed within the 80–90 °C temperature range [28]. At the same time, formation of aluminosilicate hydrates such as iron-containing siliceous hydrogarnet Ca_3_AlFe(SiO_4_)(OH)_8_ in the systems with Class C fly ash occurs at 20 °C [29].

For alkali-activated binder or geopolymer systems with Class F fly ash, the authors of [30,31] reported that more than a 10% gradual mass loss with an increase in temperature up to 800 °C was related to the dehydration of geopolymer system.

Barbosa and MacKenzie, in their study [32], reported on high-thermal resistance and excellent thermal stability of geopolymers with a potassium sialate network when exposed to temperatures of up to 1000 °C.

Regardless of the number of research studies that have been done on the effect of high temperatures on alkali-activated binder systems, a few research questions are still poorly understood as follows:

- What processes occur during structure and phase formation and transformation considering differences in chemical and mineral composition of alumosilicates?
- How are these processes correlated with the physical and mechanical performances of alkali-activated composites?

This research work addresses the evaluation of mineral phase and structure transformations in alkali-activated binders based on Class F fly ash with different chemical and mineral compositions; and studies physical and mechanical responses to a range of temperature change between 25 and 800 °C.

The outcomes of this research should help to gain a better understanding of the effect of the chemical and mineral composition of alumosilicates on chemical and structural processes that occur within a wide range of high-working temperatures.

## 2. Materials and Methods

Class F fly ash products from 3 different power plants were used in this study to prepare alkali-activated binder pastes. The physical and chemical characteristics of fly ash products are presented in Tables 1 and 2. Granulated caustic sodium hydroxide with 98% purity was used as an alkaline activator meeting Russian Standard GOST 2263-79 [33] specifications. Regular potable water was used as a mixing media to prepare alkali-activated binder pastes.

To identify the mineral composition of fly ash products, as well as reaction products and track phase transformations of alkali-activated binders exposed to high-temperatures, XRD analysis was used. The XRD patterns were produced using an X-ray station (WorkStation, Thermo Scientific, Waltham, MA, USA) ARL 9900 with filtered λ_Co_radiation. The DDM v. 1.95d software was applied for quantitative phase analysis [34]. An X-ray diagnostic of mineral phases was performed based on the diffraction database PDF-2 under the support of Crystallographica SearchMatch v 2,0,2,0 (Oxford Cryosystems, Oxford, UK) software.

The content of the vitreous phase was calculated following the method proposed by Fernandez-Jimenez etc. [35]. The total glassy content present in the fly ash was determined by subtracting all the crystalline phases from unity using 10% of TiO_2_ as an internal standard. A more detailed procedure is described by Fernandez-Jimenez etc.

The DTA/TG analysis was carried out using NETZSCH STA 449 F1 Jupiter (Erich NETZSCH GmbH & Co. Holding KG, Selb, Germany) to study the evolution in phase transformations that occur in alkali-activated binder systems. The analysis was performed in argon media with a heating rate of 10 °C/min from 25 to 800 °C. The microstructural changes in alkali-activated binders were identified and analyzed using SEM technique.

For each test, there were 2 replicates, for all analytical tests, and then the results were averaged. The analytical error is less than 5% in the case of XRF and XRD analysis, and less than 10% for PSD analysis, which is appropriate due to the multifractional nature of the analytes.

## 3. Results and Discussions

### 3.1. Structural Characterization of Fly Ash Products

The results from the XRF study for fly ash products are presented in Table 1. The studied fly ash products contain a very low amount of CaO (i.e., less than 4%), and therefore are considered to be Class F fly ash. In agreement with the classification proposed by Davidovits [18], the Si/Al oxides ratios of 1.64 and 1.76 for the fly ash products FA2 and FA3, respectively, make the materials excellent candidates for the synthesis of alkali-activated binders with thermal resistant performance. For the FA1, this ratio exceeds a value of 2, and therefore a different performance response of alkali-activated compound based on this product should be anticipated.

The LOI values in the case of coal ash products predominantly define the presence of unburned carbon. The results of XRF analysis demonstrated an elevated concentration of carbon, wherein the case of FA1 and FA2 ash products, those concentrations exceeded the limits regulated by ASTM C618. In previous research studies, the authors of [36,37] reported on the negative impact of unburned carbon as an inert component in a fly ash system, which resulted in poor alkali-activated binder structure formation, and therefore a reduction in strength performance.

The mineral composition results obtained from the XRD study together with the physical characteristics of the fly ash products are presented in Table 2.

**Figure 1:**
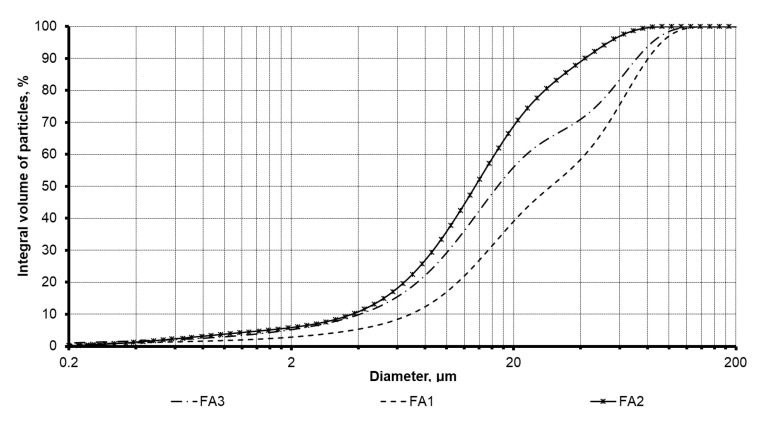
Particle size distribution of the investigated fly ash.

**Figure 2:**
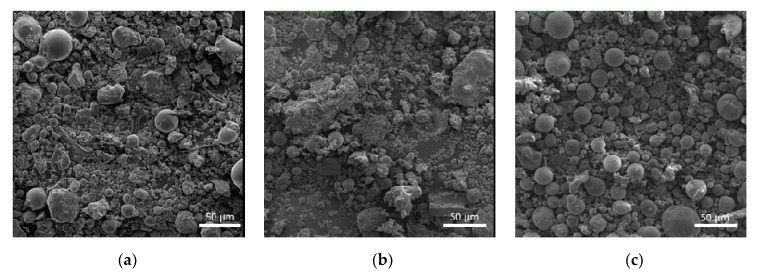
Micrographs of the fly ash products. (**a**) FA1; (**b**) FA2; (**c**) FA3.

The fly ash products dominantly contain a vitreous phase followed by mullite, quartz, and associated minerals. The studied fly ash products are represented by multifractional powders with a wide range of particle size distribution. The cumulative curves of the PSD depicted in Figure 1 are consistent with the specific surface area (SSA) results given in Table 2 for the studied fly ash. Among the fly ash products, the FA2 product is the finest material with the cumulative curve shifted towards smaller fractions with 90% of the total volume of the particles that does not exceed 45 µm limit. In addition, the SSA for this product shows the highest value of 435 m^2^/kg. At the same time, the FA1 product contains a higher percentage of coarser fractions, where 90% of the particles are less than 90 µm. This product has the lowest SSA value of 187 m^2^/kg which is explained by the presence of a large number of granular particles and fused fly ash cenospheres, as depicted in Figure 2. Prior to use, this fly ash was milled using a ball mill with uralite lining in order to increase its dispersity. The final product was as fine as 290 m^2^/kg, which was in the same range of dispersity as the other two fly ash products.

The microstructural images show that the studied fly ash products are represented by a mix of cenospheres and granular particles of alumosilicates, iron oxides, and traces of carbon (Figure 2). The FA1 and FA2 products mostly consist of granular particles and agglomerates, whereas FA3 is predominantly represented by cenospheres with a wide size range. The morphological differences in fly ash products are the result of different technologies of coal combustion used by power plants.

Earlier studies [36,37] have shown that the use of milling equipment with metallic lining for dispersion fly ash powders resulted in milling yield in the form of Fe component. When Fe reacts with water during the alkali activation process, it turns into iron hydroxide Fe(OH)_2_ with the volume 3–4.5 times larger than that of Fe. Such phase transformations unavoidably create inner pressures followed by the destruction of the matrix.

### 3.2. Mix Design and Specimens Preparation

Three alkali-activated compositions, using different fly ash products, were prepared with a constant NaO_2_/Al_2_O_3_ molar ratio. For all compositions, slump was kept constant by adding an appropriate amount of water. The mix proportions are illustrated in Table 3.

For all tests, alkali-activated binder-based specimens were prepared in cube molds of 20 × 20 × 20 mm. Then, the molded pastes were vibrated and stored at the temperature of 24 ± 1 °C for 24 h. After that, the specimens were demolded and immediately exposed to thermal treatment at 80 °C for 24 h with the following curing at room temperature for 28 days.

After this period, 50% of the specimens were exposed to thermal treatment with a gradual increase in temperature from 25 to 800 °C with the heating rate of 4 °C/min. Three specimens of each mix were tested for strength performance after temperature exposure at 25, 100, 200, 400, 600, 800 °C. The XRD and SEM analysis were performed for each composition immediatley after 28 days of curing and after 400, 600, and 800 °C thermal exposure. For the thermal resistance test, alkali-activated binder-based specimens were placed in a muffle for 15 min at each control point. The number of replicants was three for each testing point, then, the obtained data values were averaged for the analysis.

### 3.3. Thermal Effect on the Composition and Structural Changes

**Figure 3:**
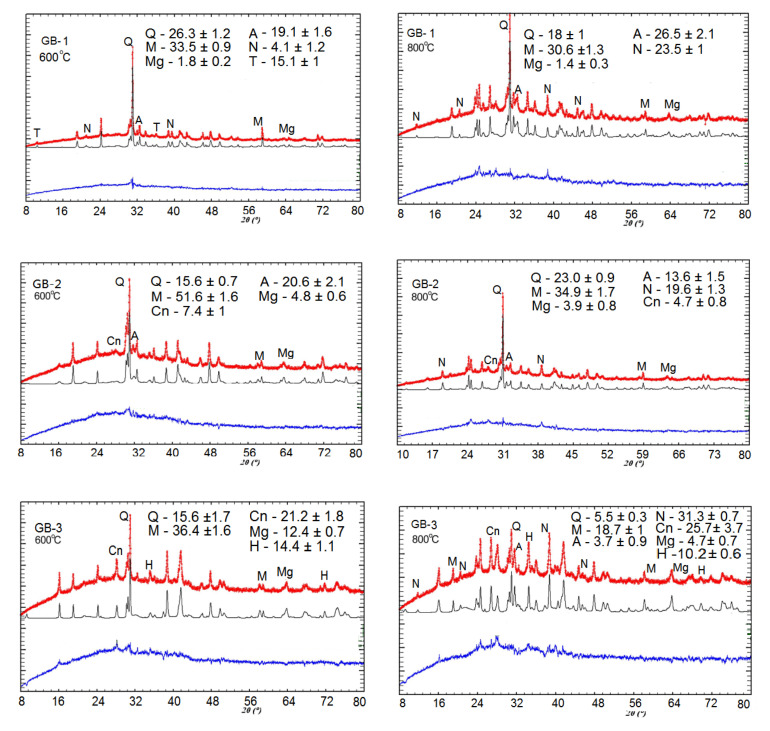
Quantitative Rietveld refinement of XRD patterns for alkali-activated binders at 600 °C and 800 °C of thermal exposure (Q, quartz; M, mullite; Mg, magnetite; H, hematite; A, anorthite; N nepheline; T, trona; Cn, cancrinite). Here, red line is for theoretical profile; black line is for experimental profile; and blue line represents the difference between the theoretical and experimental profiles.

The XRD analysis of alkali-activated binder-based specimens exposed to thermal treatment within the range of 25–800 °C (Figure 3) revealed the formation of amorphous substances and crystalline phases of Na-alumosilicates such as nepheline (Na)AlSiO_4_, anorthite Ca(Al_2_Si_2_O_8_), and cancrinite Na_7_(Al_6_Si_7_O_26_)·5H_2_O. In addition, the formation of these substances accelerates as temperature increases.

The XRD pattern of the alkali-activated binders detected the transformation of mullite and quartz, into anhydrous crystals with a network structure such as Na-alumosilicates (nepheline) and (Ca-Na)-alumosilicates (anorthite, cancrinite) at the temperature range of 25–800 °C, as depicted in Figure 3. Aluminosilicate phases cancrinite and anorthite are formed first, within a lower temperature range starting from 25 °C and higher. Further temperature increase stimulates an intensive transformation of low-temperature phases such as sodium hydrocarbonate (Na_3_(HCO_3_)(CO_3_)·2H_2_O) or trona to high-temperature crystalline mineral phases such as nepheline. Trona is a reagent used for binding sulfur trioxide in flue gas during the coal combustion process. The concurrent disappearance of peaks that define trona phases and emerging new peaks indicating nepheline presence serves as an example of such transformations. Here, for the GB-1 specimens, the formation of nepheline phase was detected at 600 °C and for GB-3 and GB-2, the XRD peaks indicating nepheline or other like phase was observed only after 800 °C of thermal exposure (Figure 3).

**Figure 4:**
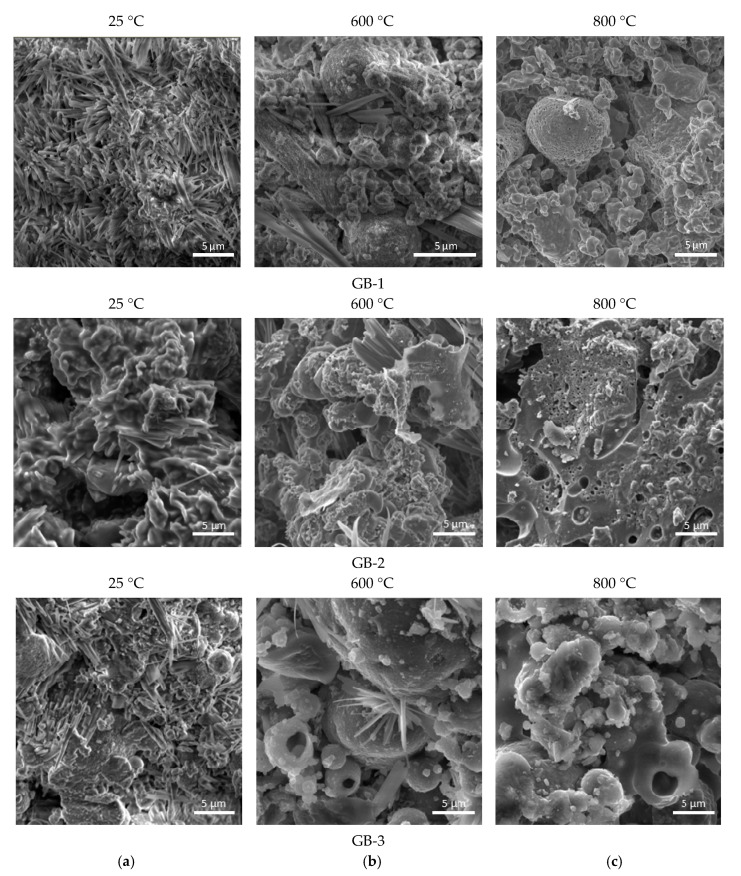
Micrographs of the alkali activated compositions after thermal treatment at different temperatures (**a**) 25 °C; (**b**) 600 °C; (**c**) 800 °C.

SEM analysis of the alkali-activated compositions, before and after thermal treatment, was performed to support the observations from the XRD study. Figure 4 presents the micrographs obtained for all geopolymers compositions, exposed to thermal treatment in the range of 400–800 °C. Microscopic study of the alkali-activated binders (Figure 4) before thermal treatment (at 25 °C) confirmed the presence of a very small portion of alkaline-alumosilicate substance in the amorphous state and a high amount of needle-like crystals of sodium carbonate hydrate formed due to the reaction of free NaOH and atmospheric CO_2_, especially for GB-1. In addition, the specimen has an incoherent and low-dense microstructure unlike the GB-2 and GB-3 specimens that respond to the lowest-strength performance before and after thermal treatment.

Among all studied alkali-activated binders, the GB-1 specimens had the highest initial content of needle-like crystals of sodium carbonate hydrate phase in the composition (Figure 4a), and with a temperature increase transformed to amorphous substance to nanosized crystalline alumosilicate phases of nepheline, as has also been reported by other authors [18,22,38]. For all alkali-activated mixes, an increase in temperature to 600 °C and 800 °C allowed for better solubility of alumosilicate component in alkaline media, and therefore the formation of an additional vitreous phase that contributed to the formation of a denser matrix. The SEM micrographs of the GB-2 and GB-3 specimens after thermal exposure at 600 °C and 800 °C revealed the presence of high-temperature nepheline phase (Figure 4b,c).

**Figure 5:**
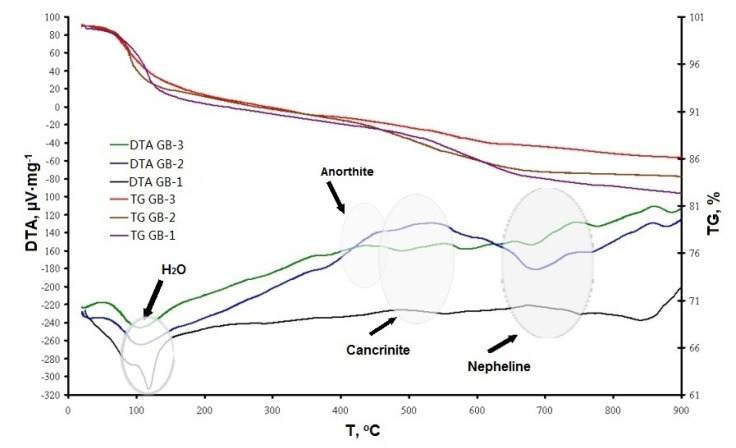
Thermogravimetric analysis of alkali-activated compositions.

The results of DTA/TG studies of all three experimental geopolymer mixes, depicted in Figure 5, showed a similar response. All compositions had an anticipated endothermic peak in the range of 100–120 °C that was responsible for the loss of chemically-unbound water. The curve for the GB-1 specimens had a more prominent mass loss in this temperature range, indicating more free water available in the system, which did not participate in chemical reactions.

The second exothermic peaks that appeared at 450–550 °C for all three curves indicated cancrinite formation for GB-1 and GB-2 specimens, as well as anorthite formation for GB-3 specimens. More pronounced peaks in these temperature intervals were detected for the GB-2 specimens. The third exothermic flux at 700–780 °C referred to the formation of the nepheline phase for all alkali-activated compositions. These data are in agreement with the XRD study (Figure 3).

### 3.4. Effect on Structural Integrity, Compressive Strength, and Surface Appearance

**Figure 6:**
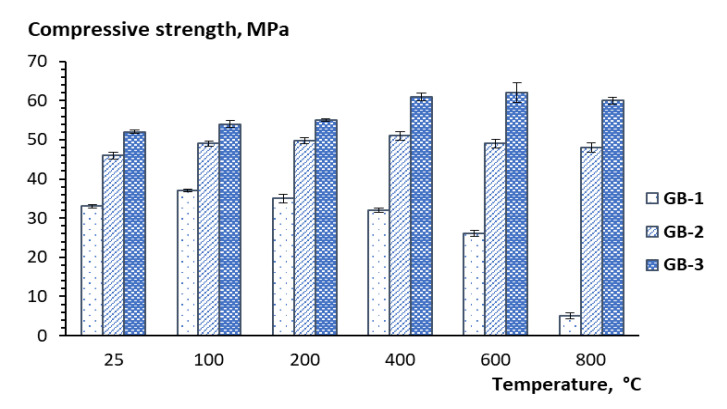
Temperature dependence of strength performance of alkali-activated binders.

**Figure 7:**
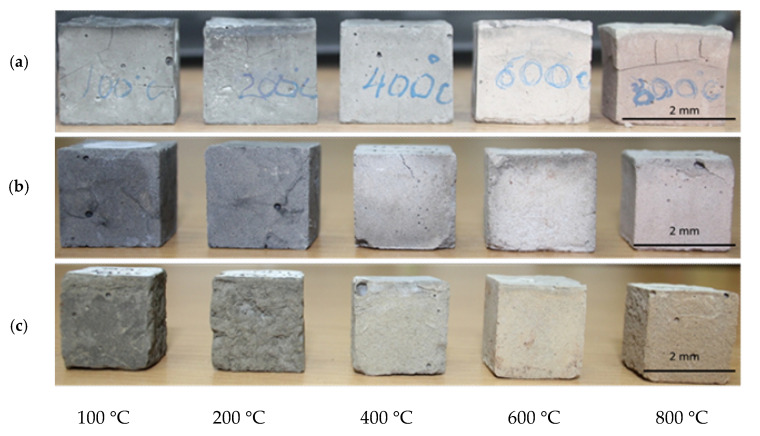
Physical configuration of the fly ash-based geopolymer specimens after thermal resistance test. (**a**) GB-1; (**b**) GB-2; (**c**) GB-3.

The compressive strength, as well as the visual structural integrity of the geopolymer compositions produced with fly ash products from different sources were evaluated after 28 days of curing, and then after thermal exposure at 100, 200, 400, 600, and 800 °C. The 28-day compressive strength results for the geopolymer compositions before thermal exposure were used as reference points in this study (Table 3). The results indicate that the thermal treatment at 100 °C somewhat improves compressive strength, which is observed for all three geopolymer compositions (Figure 6). A further temperature increase has a detrimental effect on strength with a 25% drop within the 200–600 °C temperature range and an additional 86% strength reduction within the 600–800 °C temperature range for GB-1 specimens (Figure 6). Notably, the strength reduction is accompanied by the visual disintegration of the specimens, as depicted in Figure 7a.

At the same time, the other two GB specimens did not show any effect on strength growth but rather a tendency to a reduction, up to 6%, in the case of GB-2 within the 400–800 °C temperature range or demonstrated up to 15% strength development for GB-3 within the 200–600 °C temperature range with further 2% strength reduction within the 600–800 °C temperature range (Figure 7). The structural integrity of these two specimens also looked good with a few cracks observed on the GB-2 specimens after 400, 600, and 800 °C of thermal exposure. The formation of cracks observed in the specimens was the result of thermal shrinkage [39], as well as disintegration processes related to the ongoing phase transformation. Although the specimens with visible cracks should not be subjected to compressive strength tests to evaluate mechanical performance, in this study, the specimens were tested, and the values were compared with others to estimate the residual strength after thermal exposure.

The strength performance dynamics of the studied geopolymers correlation well with mineral phase transformation that occurs with temperature increase, as seen in XRD patterns (Figure 3). An apparent drop of strength, in the case of GB-1 specimens, can be explained by the dehydration process of trona followed by the formation of nanosized crystals of nepheline from the mullite and quartz, as well as aluminosilicate vitreous phase. An observed strength reduction at a higher temperature for specimens GB-2 and GB-3 is also the result of the transformation of the mullite and quartz of fly ash to a crystalline phase of nepheline. The vitreous-crystalline phase transformation of the matrix volume changes, and therefore builds a lot of inner pressure, eventually leading to composite disintegration. At the same time, high-temperature mineral phases such as nepheline are thermally stable and contribute to matrix stability, as has been reported in earlier studies [22].

It was also observed that thermal treatment triggered a change in color of the specimens towards lighter shades (Figure 7) which could be associated with oxidation of iron and burning of carbon residuals in fly ash products [40,41].

## 4. Conclusions

In this study, an extensive analytical and experimental program was completed to obtain a better understanding of the effect of chemical and mineral composition of alumosilicates on chemical and structural processes that occur within a wide range of high working temperatures and its correlation with mechanical and physical performance of Class F fly ash-based alkali-activated binder systems. The experimental results of the study brought about several conclusions as follows:

- The XRD, SEM, and DTA/TG analyses showed that the studied geopolymers mixes exposed to high temperatures within 450–550 °C underwent a partial transformation of amorphous alumosilicate substance to the crystalline phase of anorthite Ca(Al_2_Si_2_O_8_) and cancrinite Na_7_(Al_6_Si_7_O_26_)·5H_2_O. Furthermore, in the temperature interval 600–800 °C, the rest amorphous alumosilicate substance, mullite, and quartz, as well as low-temperature phases sodium hydrocarbonate (Na_3_(HCO_3_)(CO_3_)·2H_2_O) or trona in the case of GB-1 transforms into anhydrous crystals with network structure such as Na-alumosilicates nepheline.
- The presence of 15% of trona in the GB-1 specimens demonstrates a considerable detrimental effect and leads to up to 86% of strength loss within the temperature range of 600–800 °C when trona undergoes the dehydration process. The other two specimens GB-2 and GB-3, which did not contain trona in the system were able to develop strength with a temperature increase up to 400 °C and 600 °C, followed by a strength loss within the temperature ranges of 400–800 °C and 600–800 °C, respectively.
- A strength loss up to 15% in the GB-2 and GB-3 within the 400–800 °C temperature range is the result of volume change, which occurs during the transformation of mullite and quartz of fly ash to crystalline phases such as anorthite, cancrinite, and nepheline, leading to inner pressure buildup and further matrix disintegration.

The overall outcomes of the study are as follows:

- High temperatures facilitate solubility of alumosilicate components in alkaline media, resulting in synthesis of additional vitreous phase and formation of nanosized crystals of high-temperature nepheline. These concurrent processes both contribute to formation of a high-temperature stable nepheline phase, densification of the alkali activated matrix, and an increase in strength response of alkali activated binders.
- The presence of an unfavorable carbonate mineral phase trona in the alkali activated binder system is the result of the reaction between CO_2_ and “free” alkaline component which did not react with alumosilicates.
- Low solubility of alumosilicates in alkaline media, leaving a big quantity of unreacted components, leads to a poor cross-linking of -Si-Al- chains, creating a low degree of geopolymerization that results in an incoherent matrix.

## Figures and Tables

**Table 1:** Chemical composition of the fly ash products.

Oxide Content (WT. %)												
Fly Ash ID	LOI	SiO_2_	Al_2_O_3_	Fe_2_O_3_	CaO	TiO_2_	K_2_O	Na_2_O	MgO	SO_3_	P_2_O_5_	Si/Al Ratio
FA1	6.1	58. 6	24.3	5.3	2.3	0.9	0.7	0.5	0.5	0.3	0.1	2.41
FA2	5.2	57.4	32.7	0.5	0.4	0.1	0.4	1.2	2.2	-	0.6	1.64
FA3	1.3	45.6	27.8	15.6	3.6	1.2	1.7	1.0	1.3	1.3	0.4	1.76

**Table 2:** Mineral and physical characteristics of the fly ash products.

Fly Ash ID	Parameter		Mineral Composition, %					
	Spec. Gravity	Specific Surface Area, m^2^/kg	Quartz	Mullite	Anorthite	Magnetite	Hematite	Vitreous Phase
FA1	1.87	290	10.7	23.5	4.3	1.0	-	60.5
FA2	1.80	435	9.3	18.7	-	1.9	-	70.1
FA3	1.69	257	6.4	13.5	-	7.2	4.5	68.4

**Table 3:** Mix design and compressive strength of fly ash-based alkali-activated compositions.

Specimen	FA, %	NaOH, %	Water, %	Na_2_O/Al_2_O_3_ Molar Ratio	Compressive Strength, MPa
GB-1	66.5	10.5	23.0	0.75	34.1
GB-2	63.3	8.0	28.7	0.75	45.2
GB-3	68.8	12.6	18.5	0.75	50.3
